# Sociality does not predict signal complexity in response to playback in apteronotid weakly electric fishes

**DOI:** 10.1007/s00265-025-03619-y

**Published:** 2025-07-10

**Authors:** Megan K. Freiler, G. Troy Smith

**Affiliations:** 1 Department of Biology, Indiana University, Bloomington, IN USA; 2 Center for the Integrative Study of Animal Behavior, Indiana University, Bloomington, IN USA

**Keywords:** Chirping, Electrocommunication, Signal structure, Social complexity

## Abstract

Evolution of signal structure should be influenced by the social environment in which signals are sent and received. The “social complexity hypothesis” for communicative complexity postulates that individuals living in complex social groups require complex signals to communicate effectively. This hypothesis has been supported in many vocalizing animals. In other systems and modalities, however, relationships between sociality and signal complexity are less clear. Weakly electric knifefishes represent a novel model for investigating the relationship between signal structure and social structure. South American apteronotid knifefish continuously produce quasi-sinusoidal electric organ discharges (EODs) from their tail to communicate species, sex, and status. Fish also rapidly modulate EOD frequency to produce chirps, which coordinate agonistic and courtship interactions. Sociality and chirp structure vary extensively across apteronotids. Here, we quantified chirp complexity and variation in chirp structure across six species that vary in group size: three territorial species (*Apteronotus albifrons*, *Parapteronotus hasemani*, and *‘Apteronotus’ bonapartii*), two gregarious species (*Adontosternarchus devenanzii* and *Adontosternarchus balaenops*), and a semi-social species (*Apteronotus leptorhynchus*). Chirp complexity and variation in chirp structure differed substantially across species but were unrelated to sociality. Within the *Apteronotus* and *Adontosternarchus* species pairs, one species produced complex chirps, whereas the other produced relatively simple chirps. Thus, neither phylogenetic relatedness nor social structure explained variation in chirp structure or complexity in the species we examined.

**Supplementary Information:**

The online version contains supplementary material available at 10.1007/s00265-025-03619-y.

## Significance statement

The relationship between sociality and signal complexity has never been tested in the electric signaling modality. Electric fishes rely primarily on their electric sense to communicate and have extensive species variation in both social and signal structure, making them an ideal model in which to investigate how social behavior and signals covary. Apteronotid weakly electric fishes produce simple, easily quantifiable electrocommunication signals called chirps during social interactions. Group size was not associated with chirp complexity or variation in chirp parameters across six apteronotid species with a range of social structures. These findings highlight the need to investigate the relationship between social structure and signal design across multiple species and modalities to determine whether more complex social environments universally require more complex signaling regimes.

**Supplementary Information:**

The online version contains supplementary material available at 10.1007/s00265-025-03619-y.

## Introduction

Communication is fundamental for coordinating social interactions and is an important target of selection in the evolution of social behavior. The evolution of signal structure and complexity should, therefore, be tightly linked to social structure. The “social complexity hypothesis” for communicative complexity posits that more complex social structures necessitate more varied signaling systems (Freeberg et al. 2012). If social interactions involve more individuals and/or social networks are more intricate, more information may be required to resolve and respond to social signals appropriately. Species that have more frequent social interactions might also be expected to rely on more signals or more varied signal types to coordinate their more numerous encounters. The evolutionary and physiological mechanisms driving relationships between social and signal variation are, however, often unclear (Freiler and Smith 2023). Social complexity predicts signal complexity in many taxa (Blumstein and Armitage 1997; McComb and Semple 2005; May-Collado et al. 2007; Leighton 2017; Knörnschild et al. 2019; Fichtel and Kappeler 2022), but this positive relationship between social and signal complexity is not universal. In other systems, social structure is unrelated to signaling behavior (Ord and Garcia-Porta 2012; Peckre et al. 2019; Warren et al. 2021). Whether social complexity requires signal complexity or vice versa across animal systems is, therefore, not certain (Peckre et al. 2019).

While historically, the social complexity hypothesis has focused primarily on vocal communication, recent studies have also found positive relationships between social complexity and olfactory and visual signal complexity (delBarco-Trillo et al. 2012; Wittwer et al. 2017; Roberts and Roberts 2020; Baeckens and Whiting 2021; Fichtel and Kappeler 2022; Lin et al. 2024). Continuing to expand investigations beyond the acoustic modality will help clarify if social complexity requires signal complexity across modalities. Many animals also rely on multiple sensory channels for communication, and sometimes multimodal signals are present in a single display, which can complicate quantifying signal complexity (Thompson et al. 2008; Ronald et al. 2018, 2020). Social complexity might only predict signal complexity in one modality or it could be positively correlated with communicative complexity across signal modalities (Martins et al. 2018; Fichtel and Kappeler 2022). Relationships between social and signal complexity may be easier to identify in tractable systems that largely rely on signals in a single modality whose parameters and complexity are simple to quantify.

The electric communication signals of electric fishes provide a novel opportunity to examine relationships between social and signal complexity across species. South American apteronotid fish produce a weak electric organ discharge (EOD) from their tail. Fish each produce a stable EOD frequency (EODf) that varies across species and with sex in sexually dimorphic populations (Ho et al. 2013; Smith 2013). While electric fishes can use visual signals and some species use acoustic signals (Crawford et al. 1986; Stamper et al. 2012; Stevens et al. 2013; Van Nynatten et al. 2019), apteronotid knifefishes do not produce sound and have poor color vision and visual spatial acuity (Takiyama et al. 2015; Liu et al. 2016), and thus, rely predominantly on EODs to navigate their surroundings and for communication. They also modulate their baseline EODf during agonistic and courtship interactions to produce signals called chirps. Chirp structure, rate, and function vary across species and sex (Turner et al. 2007; Smith 2013). While a few species have discrete chirp types, variation in chirp structure is typically graded within species (Turner et al. 2007; Smith 2013), which makes quantifying a repertoire size difficult. Chirps can vary in duration, frequency modulation, slope, and complexity, however. Chirp complexity can be defined as the number of frequency changes during the chirp (Kershenbaum et al. 2018). Both behavioral responses and sensory encoding strategies for chirps vary between species and across chirp structure, suggesting fish can discriminate between chirps that differ in structure and complexity (Marsat et al. 2012; Petzold et al. 2016; Allen and Marsat 2018; Dillon-Seeger 2019).

Apteronotids also vary extensively in social behavior. Some species are highly territorial and aggressive, whereas other species are highly affiliative (Hagedorn and Heiligenberg 1985; Hupé and Lewis 2008; McNeil 2014; Allen 2019). While group size alone cannot capture all aspects of a species’ social behavior, it is currently the best proxy for classifying sociality in electric fishes and predicts agonistic behavior. Social complexity can be treated as a categorical variable in tests of the social complexity hypothesis (May-Collado et al. 2007; Wittwer et al. 2017; Baeckens and Whiting 2021). Furthermore, any quantitative measure of group size only represents the species average and would not capture within species variation in grouping behavior. We, therefore, categorized apteronotid species as either ‘territorial’, ‘semi-social’, or ‘gregarious’. Territorial electric fish species are often found alone in the field and in the lab (Stamper et al. 2010; Carlson 2016). Similarly, in several species of apteronotids, including *Apteronotus albifrons*,* Parapteronotus hasemani*, and *“Apteronotus” bonapartii*, both sexes are highly aggressive, spend most of their time alone in shelters, and attack each other, often to the point of injury, when housed in groups or when placed in dyadic interactions (personal observation). Semi-social species form small groups of 2–4 fish, like *Apteronotus leptorhynchus* (Stamper et al. 2010). *A. leptorhynchus* also form dominance hierarchies. Mature males are often more aggressive, can be found with several females and immature fish, and preferentially occupy shelter tubes alone compared to females (Hagedorn and Heiligenberg 1985; Dunlap and Oliveri 2002; Raab et al. 2021; Osorio Ospina 2023). In contrast, *Adontosternarchus* species are highly gregarious and preferentially group together in a single large shelter structure when initially separated in an artificial habitat containing multiple interconnected tanks each with more than one shelter (Steinbach 1970; McNeil 2014). *Adontosternarchus* are also morphologically sexually monomorphic, prefer open water, exhibit very little aggression when placed in dyads, and display signs of stress (high reactivity, highly perseverative movements) when housed alone (personal observation).

Apteronotid species diversity in both chirp structure and in social behavior make knifefishes an ideal model in which to test the social complexity hypothesis in a new modality. While apteronotids vary in sociality and chirping behavior, it is unclear whether variation in group size is related to variation in chirping across species. To test the social complexity hypothesis in apteronotids, we quantified chirp complexity and variation in chirp structure in six species that vary in group size: territorial *A. albifrons*,* P. hasemani*, and *‘A.’ bonapartii;* gregarious *Adontosternarchus devenanzii* and *Adontosternarchus balaenops;* and semi-social *A. leptorhynchus.* If the social complexity hypothesis applies in apteronotids, we expected to see the most complex, variable chirps in *A. devenanzii* and *A. balaenops*, somewhat less chirp complexity and variability in *A. leptorhynchus*, and the least variable and most simple chirps in *A. albifrons*,* P. hasemani*, and *‘A.’ bonapartii*.

## Materials and methods

### Animals

Fish were sourced from commercial fish suppliers [*A. albifrons* and *A. leptorhynchus* (Ruinemans, Miami, FL, USA), *P. hasemani* (Ornamental Amazon Fish Aquarium, SAC, Iquitos, Peru), *A. balaenops* and *‘A.’ bonapartii* (Riverland, Iquitos, Peru), *A. devenanzii* (Rose Tropical Fish, Miami, FL, USA)] and were recorded in the laboratory at Indiana University, Bloomington. Two *‘A.’ bonapartii* individuals were collected from tributaries of the Solimoes River and were recorded at the Instituto Nacional de Pesquisas da Amazonia (INPA) in Manaus, Brazil. Fish were housed in water maintained at a temperature between 26–28ºC, a conductivity between 100 and 600 µS cm^−1^, and a pH between 5.5 and 6.5. Fish were fed live blackworms or frozen bloodworms three times a week.

### Recordings

Archived recordings collected from previous playback studies were analyzed for the six species of interest: *A. leptorhynchus* (*N* = 14; all M) (Smith and Combs 2008), *A. albifrons* (*N* = 13; 6 M, 6 F, 1 unknown sex) (Ho et al. 2013), P. *hasemani* (*N* = 10; 6 M, 4 F) (Petzold and Smith 2016), *‘A.’ bonapartii* (*N* = 8; 4 M, 1 F, 3 unknown sex) (Turner et al. 2007; Ho et al. 2010), A. *devenanzii* (*N* = 15; 12 M, 3 F) (Zhou and Smith 2006), and *A. balaenops* (*N* = 3, unknown sex) (Turner et al. 2007). Additionally, unpublished *A. balaenops* (*N* = 11, unknown sex) recordings collected in 2014 and 2019 were analyzed. In studies that used hormone or drug treatments (Smith and Combs 2008; Ho et al. 2013; Petzold and Smith 2016), only baseline recordings from fish that had not yet received a hormone or drug treatment were used. Recordings with an average > 3 Hz of noise in the frequency trace were also excluded from the analysis.

Chirping behavior was recorded in *A. balaenops* and was collected previously from the other species by using a chirp playback paradigm that has been described before (Kolodziejski et al. 2005; Petzold and Smith 2016; Petzold et al. 2018). Fish were either enclosed in a shelter tube or placed in a mesh net inside a darkened tank. A pair of carbon electrodes placed in front of the fish’s head and behind its tail recorded the fish’s EOD and chirping. Stimulus signals were generated in audio software (CoolEdit Pro, Syntrillium; Phoenix, AZ, USA) and were presented via a sound card connected to a pair of carbon electrodes placed on the left and right side of the fish. The stimulus strength was calibrated halfway between the playback electrodes to a root-mean-square amplitude that was of similar intensity to the EOD of an average sized fish of each species (typically 0.6–1.5 mV cm^−1^). Following a 15–60 min acclimation period and a four-minute baseline recording, each fish was presented with a series of five sinusoidal voltage stimuli with frequencies that ranged between − 150 Hz and + 150 Hz relative to the fish’s own EODf. Recordings were four minutes long with a one-minute pre-stimulus period, a two-minute playback, and a one-minute post-stimulus period. Recordings with each of the five different playback stimuli were separated by 10 min to reduce habituation. Recordings were sampled at 44.1 kHz and 16-bit resolution.

### Chirp counting

While the experimenters were not blind to the species in each recording, all chirps were counted automatically with the same algorithm across species. Files were processed and chirps were identified using custom programs (Brian Nelson, University of Oregon; Eugene, OR, USA) in Igor Pro (WaveMetrics; Portland, OR, USA). Details have been described in previous studies (Kolodziejski et al. 2005; Ho et al. 2010). Contamination from the playback stimulus was removed by subtracting a scaled and phase shifted copy of the playback from the original recording. EODf was calculated by using an autocorrelation algorithm on 6-ms windows that slid 2 ms/iteration, resulting in a sampling rate of 500 Hz for EODf. Chirps were counted when EODf was at least 3 Hz above baseline EODf for a minimum of 3 ms in most cases. The baseline EODf was calculated using the 3 s before and after a chirp occurred. In cases when the baseline EODf was unstable around chirps, the EODf baseline was reduced to 1–2 s. For *P. hasemani*, an EODf baseline of 10 s was used to ensure an accurate baseline measure around their long duration chirps. A negative frequency change had to be at least 2 Hz below baseline to be counted as a chirp undershoot. The minimum interchirp interval was set at 100 ms. The start and end times of each chirp were marked when EODf was within 1 Hz of baseline for all species except *P. hasemani*. For the exceptionally large chirps of *P. hasemani*, a 3 Hz threshold was used to mark the beginning and end of chirps because EODf did not always return to within 1 Hz of baseline. To ensure each chirp was counted and quantified appropriately, frequency traces were also visually inspected. Chirps were excluded if they were surrounded by noise, or if a well-defined end time could not be identified.

### Complexity analysis

**Fig. 1:**
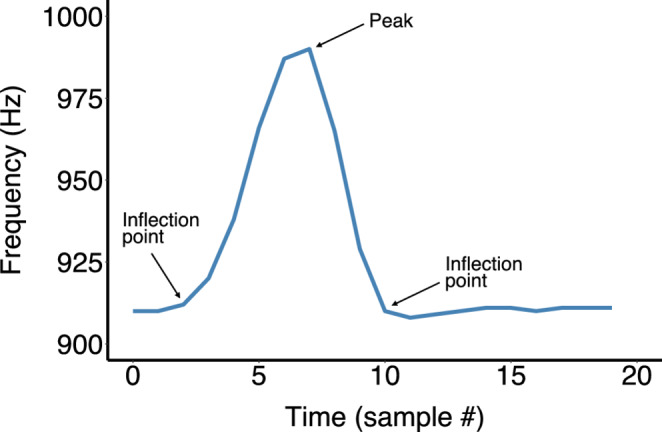
Example chirp with peaks and inflection points defined. Each time step represents 2 ms

While the experimenters were not blind to the species, complexity was quantified with the same algorithm across species. We used the number of peaks/troughs and inflection points to measure complexity, because electric fish have continuous graded variation in chirp structure (Kershenbaum et al. 2018). The number of peaks, troughs, and inflection points was counted in MATLAB R2021a (MathWorks, Natick, MA, USA) by using an algorithm based on the methods of Kershenbaum et al. 2018 (scripts available upon request). Igor binary files were imported into MATLAB using the IBWread function (Bialek 2022). The extremely large chirps of *P. hasemani* often cause EOD amplitude to drop close to zero, which makes EODf measurements unreliable. Accordingly, in *P. hasemani* recordings, EODf was fixed at the last reliable frequency measurement during the portion of chirps where EODf amplitude was less than 15% of the baseline amplitude (Turner et al. 2007). Peaks and troughs were identified as events where the first derivative of the EODf changed signs from positive to negative or from negative to positive, respectively (Fig. 1). Inflection points were identified as events in which the second derivative changed signs (Fig. 1). Peaks and troughs were only included if EODf was at least 5 Hz different from baseline EODf, and inflection points were only included if the rate of EODf change was greater than 1 Hz/ms. After these criteria were met, there were two ways a peak, trough, or inflection point could be counted. (**1)** When comparing one sample away from the point of interest, the frequency change in at least one direction had to reach a threshold of 5 Hz for peaks/troughs or the rate of frequency change in at least one direction had to reach a threshold of 4 Hz/ms for inflection points. **(2)** When comparing two samples away from the point of interest, the frequency change in at least one direction had to reach a threshold of 2 Hz for peaks/troughs or the rate of frequency change had to reach a threshold of 1.5 Hz/ms for inflection points. For this second method of inclusion, unless a peak, trough, or inflection point was within 2 samples of the start or end of a chirp, changes in EODf or the derivative of EODf also had to be sustained for a minimum of two samples in at least one direction for a peak, trough, or inflection point to be counted. These criteria prevented the algorithm from incorrectly detecting transient noise as a peak, trough, or inflection point.

Once peaks, troughs, and inflection points were counted, a subset of chirps was used for further analysis. Each fish was only represented by a maximum of 10 chirps to reduce weighting by any one individual. These 10 chirps were chosen to represent the range of complexity in the chirps produced by each fish. Peaks/troughs and inflection points of each chirp were added together for a total complexity score. Chirps from each fish were then sorted from least to most complex based on this score. Ten chirps evenly spaced across this distribution were selected to represent the range of complexity. If fish had fewer than 10 chirps, then all chirps were included.

### Statistics

All statistical analyses were completed in R v 4.4.1 (R Core Team 2024) and plotted with *ggplot2* (Wickham 2016). Species differences in the number of peaks and inflection points were assessed using Kruskal-Wallis tests because data were not normally distributed. Post hoc pairwise tests were done using Wilcoxon rank-sum tests with Benjamini-Hochberg correction for multiple comparisons (Benjamini and Hochberg 1995; Benjamini and Yekutieli 2001). To evaluate chirp structure variation and clustering within and across individuals in each species, a principal components analysis (PCA) was run using peaks, inflection points, positive frequency modulation (FM), negative FM, chirp duration, slope of the frequency increase, and slope of the frequency decrease. Positive FM was calculated by Igor as the difference between the maximum EODf during the chirp and baseline EODf. Some chirps had negative undershoots at the end of the chirp. Negative FM was calculated by Igor as the difference between the minimum EODf and the baseline EODf. Slope of the frequency increase or decrease was calculated as the positive FM divided by the time of the maximum FM of the chirp minus the start or end time of the chirp, respectively. To ensure an equal number of chirps from each species was used in the PCA, only fish with at least 10 chirps were included. *‘A.’ bonapartii* only had six fish that met this criterion, so six fish were chosen randomly from the other species to include in the PCA.

## Results

### Chirp structure and complexity varied across species

**Fig. 2:**
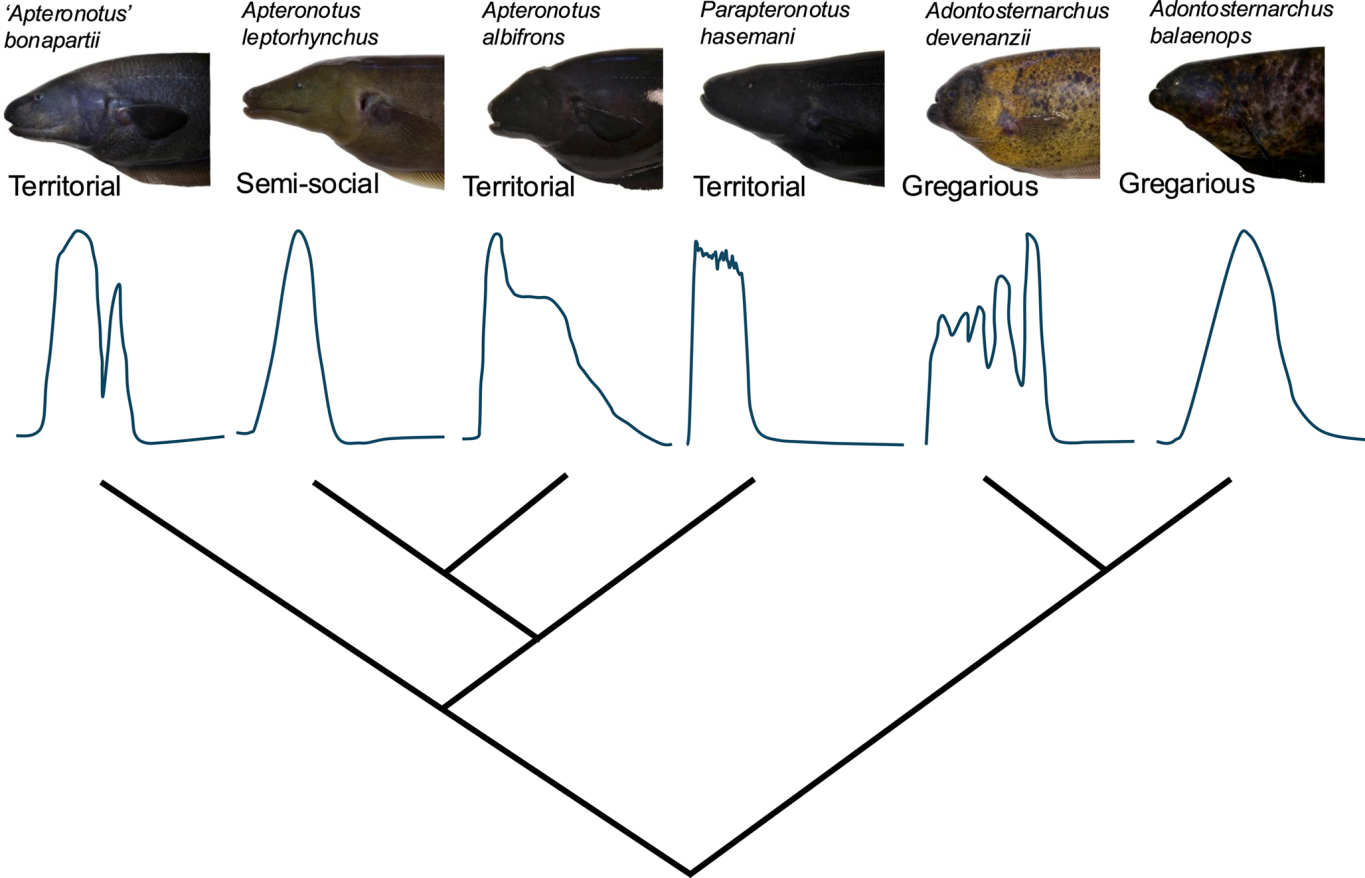
Representative chirps from each species. Blue traces represent EODf versus time during a representative chirp. Chirp FM and duration not to scale. Phylogeny based on Smith et al. 2016 and Tagliacollo et al. 2016. FM = frequency modulation, EODf = electric organ discharge frequency

**Fig. 3:**
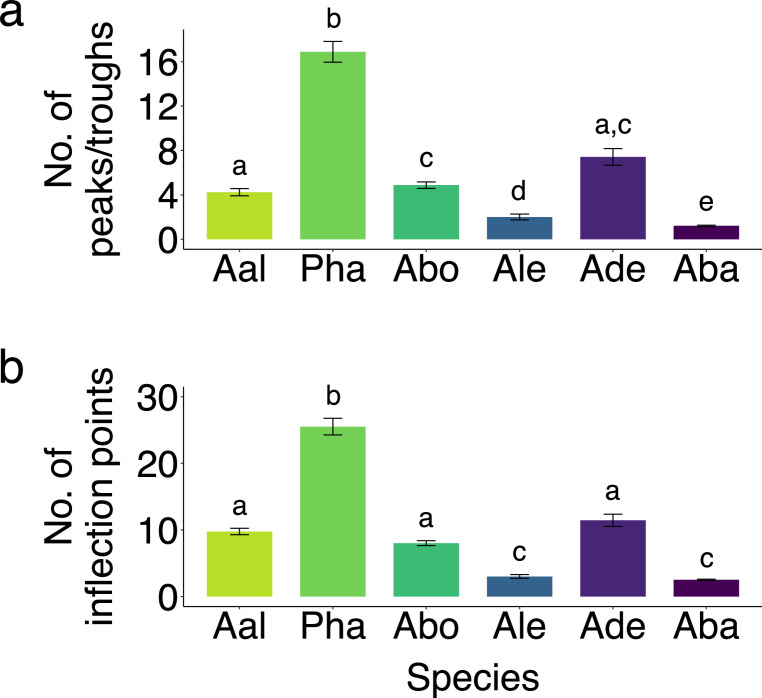
Group size did not predict signal complexity. The number of **(a)** peaks (and troughs) and **(b)** inflection points in chirps varied across species but did not map onto sociality or genus. Territorial species are represented in green, semi-social in blue, and gregarious in purple. *P. hasemani* (Pha, *N* = 10) had significantly more complex chirps than every other species. *A. albifrons* (Aal, *N* = 13), *‘A.’ bonapartii* (Abo, *N* = 8), and *A. devenanzii* (Ade, *N* = 15) had more complex chirps on average than *A. leptorhynchus* (Ale, *N* = 14) and *A. balaenops* (Aba, *N* = 14). Letters denote significant pairwise differences from Wilcoxon rank-sum tests

Chirp parameters, including positive FM (PosFM), duration, negative FM (NegFM), slope of the frequency increase (Slopeup), slope of the frequency decrease (Slopedown), peaks (and troughs), and inflections points (IPs), varied across species (Table 1). Chirp complexity varied significantly across species (Fig. 2) when determined by both number of peaks (Kruskal-Wallis test: χ^2^ = 337.94, *P* < 0.001, Fig. 3a) and number of inflection points (Kruskal-Wallis test: χ^2^ = 410.68, *P* < 0.001, Fig. 3b). Complexity was unrelated to species’ social structure or genus (Fig. 3). *P. hasemani* had the longest, highest frequency, and most complex chirps relative to all other species (Table 1; Fig. 3). *A. albifrons*, *‘A.’ bonapartii*, and *A. devenanzii* had more complex chirps than *A. leptorhynchus* and *A. balaenops* (Fig. 3). When chirp complexity was corrected for chirp duration, *P. hasemani* had the least complex chirps while *‘A.’ bonapartii* had relatively short, but highly complex chirps. The other four species had intermediate, but similar chirp complexities when peaks/troughs and IPs were normalized to duration (Fig. S1). Chirp complexity still did not map onto species’ social structure or genus with chirp complexity normalized by chirp duration. Chirp duration could be considered an important component of complexity, so we primarily discuss results without normalization.

**Fig. 4:**
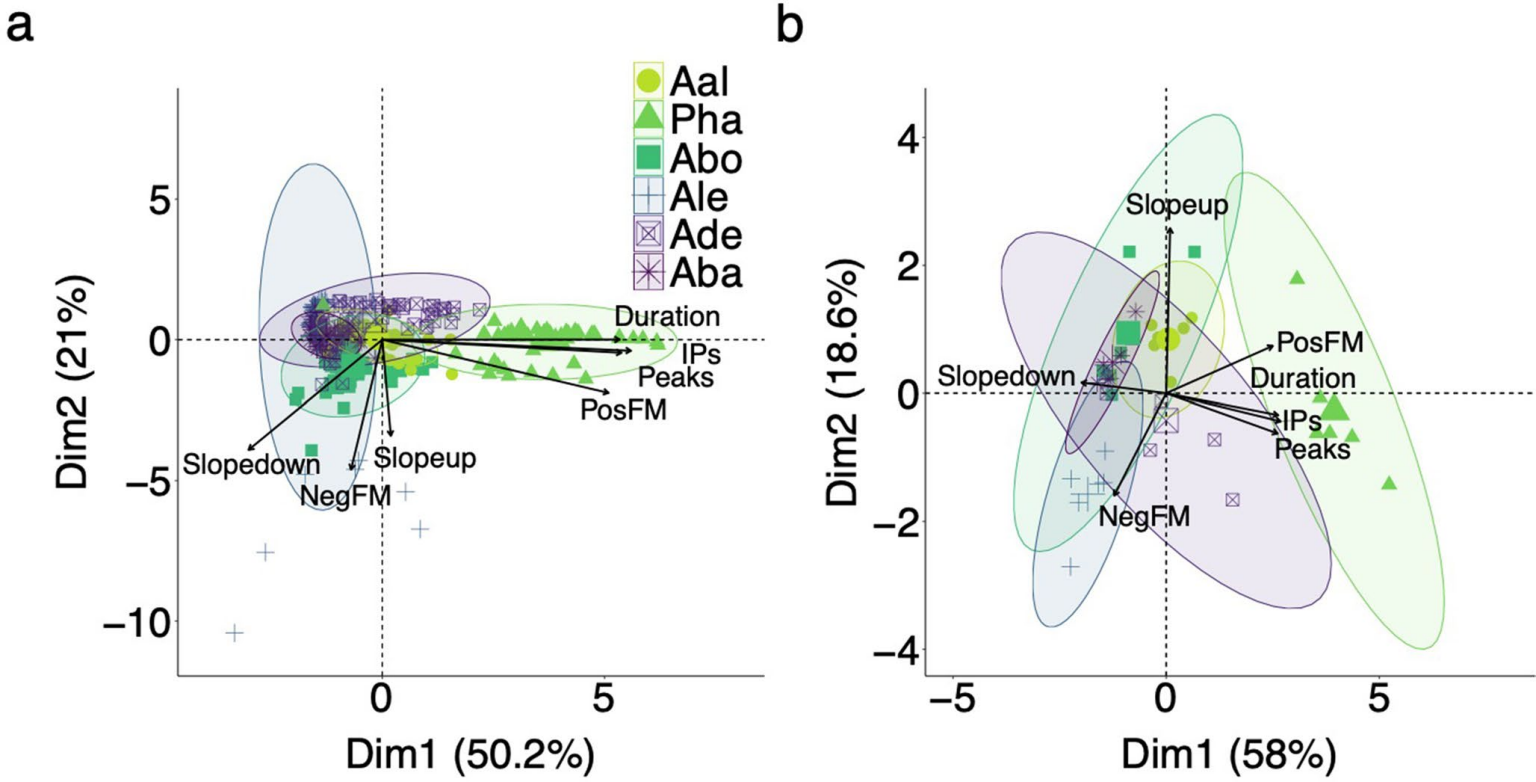
Sociality was not related to signal variation. Variation in signal parameters was largely overlapping in PCA space across species and was unrelated to sociality. Territorial species are represented in green, semi-social in blue, and gregarious in purple. **(a)** Each point represents a chirp and the larger point at the center of the ellipse represents the species average. *P. hasemani* (Pha), *A. leptorhynchus* (Ale), and *A. devenanzii* (Ade) had larger distributions than *A. albifrons* (Aal), *‘A.’ bonapartii* (Abo), and *A. balaenops* (Aba) (*N* = 60 chirps/species). **(b)** Each point represents an individual and the larger point at the center of the ellipse represents the average across individuals (*N* = 6 fish/species). Pha, Ade, and Abo had large distributions, followed by Aal and Ale. Aba exhibited very little individual variation. Vectors represent the principal component loadings and ellipses represent 95% CIs

There was a male sampling bias in three of the six species: *A. leptorhynchus*,* ‘A.’ bonapartii*, and *A. devenanzii*. All *A. leptorhynchus* included in this data set were male, so we did not assess sex differences in chirp structure or complexity in this species. Only one female with three chirps was included in the *‘A.’ bonapartii* sample, so we did not run statistical analyses to determine sex differences in complexity in this species. This female, however, had similar chirp parameter values as the males, but a lower number of peaks/troughs and inflection points (Table S1). Three females with a total of 22 chirps were included in the *A. devenanzii* sample. Male *A. devenanzii* had chirps with longer durations and more FM (Table S1) and had a higher total complexity score (sum of peaks, troughs, and inflection points) than females (Mann-Whitney U: *W* = 370.5, *P* < 0.01). The *A. albifrons* sample included an equal number of males and females. Chirp parameter values were comparable between males and females (Table S1), and we found no significant difference in the total complexity score between the sexes (Mann-Whitney U: *W* = 1379.5, *P* = 0.23). The *P. hasemani* sample included four females and six males, and total complexity was significantly higher in male chirps (t-test: *t*(97.1)=−4.11, *P* < 0.01). Because PosFM was similar between the sexes in *P. hasemani*, this difference was likely driven by much longer chirp durations in males (Table S1). Sex was unknown in *A. balaenops* because unlike the other species, they are sexually monomorphic both morphologically and in their EOD parameters (Zhou and Smith 2003). However, chirp complexity varied little across individuals in *A. balaenops* (Fig. 4).

**Table 1:** Chirp parameters

Species	PosFM (Hz)	Duration (s)	NegFM (Hz)	Slopeup (Hz ms^−1^)	Slopedown (Hz ms^−1^)	Peaks/ troughs	IPs
*P. hasemani*	561.6 ± 8.9	0.83 ± 0.04	N/A	16.4 ± 1.04	1.05 ± 0.11	16.9 ± 0.93	25.5 ± 1.24
*A. leptorhynchus*	99.1 ± 6.7	0.04 ± 0.002	17.9 ± 2.9	8.8 ± 0.68	10.7 ± 0.80	2.0 ± 0.26	3.0 ± 0.29
*A. albifrons*	228.0 ± 5.9	0.13 ± 0.004	3.1 ± 0.11	20.7 ± 0.67	2.6 ± 0.13	4.2 ± 0.33	9.8 ± 0.48
*‘A.’ bonapartii*	263.4 ± 11.9	0.05 ± 0.003	4.4 ± 0.27	22.6 ± 0.94	13.5 ± 1.03	4.9 ± 0.29	8.0 ± 0.36
*A. devenanzii*	164.3 ± 4.8	0.16 ± 0.01	2.9 ± 0.36	8.3 ± 0.67	3.1 ± 0.39	7.4 ± 0.75	11.5 ± 0.93
*A. balaenops*	172.9 ± 8.3	0.03 ± 0.002	N/A	15.6 ± 0.58	10.4 ± 0.41	1.2 ± 0.07	2.5 ± 0.09

Mean ± SEM for several chirp parameters across species. *N* = number of chirps. *P. hasemani* (*N* = 100), *A. leptorhynchus* (*N* = 140), *A. albifrons* (*N* = 123), *‘A.’ bonapartii* (*N* = 67), *A. devenanzii* (*N* = 97), and *A. balaenops* (*N* = 114). Only some chirps had a NegFM: *P. hasemani* (*N* = 0), *A. leptorhynchus* (*N* = 80), *A. albifrons* (*N* = 30), *‘A.’ bonapartii* (*N* = 36), *A. devenanzii* (*N* = 5), and *A. balaenops* (*N* = 0). IP = inflection point, FM = frequency modulation

### Species variation in chirp structure was unrelated to sociality

To examine variation in chirp structure across individuals in each species, peaks (and troughs), IPs, duration, PosFM, NegFM, Slopeup, and Slopedown were analyzed using principal components. Two components explained 71.2% of the total variance (Fig. 4a). Dim1 explained 50.2% of the variance and loaded primarily with duration, PosFM, peaks, and inflection points, while Dim2 explained 21% of the variance and loaded primarily NegFM and Slopeup. Slopedown loaded on both dimensions. Species distributions were largely overlapping. *P. hasemani*, *A. leptorhynchus* and *A. devenanzii* had the greatest variation in chirp structure, followed by *‘A.’ bonapartii*. Specifically, *P. hasemani* and *A. devenanzii* showed higher standard deviation in Dim1 relative to the other species, while *A. leptorhynchus* had a very high standard deviation in Dim2 (Table 2). *‘A.’ bonapartii* showed moderate standard deviations in both dimensions, followed by *A. albifrons*. *A. balaenops* had little variation in chirp structure.

To analyze individual variation in chirp structure across species, the same parameters as above were analyzed using principal components but with the parameters of each individual’s chirps averaged first. Two components explained 76.6% of the total variance (Fig. 4b). Dim1 explained 58% of the variance and loaded primarily with duration, peaks, IPs, and Slopedown, while Dim2 explained 18.6% of the variance and loaded primarily with NegFM and Slopeup. PosFM loaded on both dimensions. Species distributions were largely overlapping with *P. hasemani*,* ‘A.’ bonapartii*, and *A. devenanzii* having the greatest individual variation in chirp structure, followed by *A. albifrons* and *A. leptorhynchus*. *A. balaenops* had very little individual variation in chirp structure. Standard deviations between each dimension were roughly equivalent in *A. albifrons* and *A. balaenops*. *A. devenanzii* had more variation in Dim1 while *P. hasemani*,* A. leptorhynchus*, and *‘A.’ bonapartii* exhibited more variation in Dim2 (Table 2).

**Table 2:** Means and standard deviations of PCA Dim1 and Dim2

	Dim1 mean	Dim1 SD	Dim2 mean	Dim2 SD
**All chirps**				
*P. hasemani*	3.650	1.191	−0.076	0.535
*A. leptorhynchus*	−1.454	0.527	0.104	2.452
*A. albifrons*	−0.113	0.546	0.063	0.404
*‘A.’ bonapartii*	−0.660	0.644	−0.927	0.723
*A. devenanzii*	−0.165	1.034	0.692	0.668
*A. balaenops*	−1.258	0.314	0.145	0.332
**Individuals averaged**				
*P. hasemani*	3.940	0.760	−0.273	1.096
*A. leptorhynchus*	−1.838	0.381	−1.576	0.611
*A. albifrons*	0.050	0.387	0.843	0.360
*‘A.’ bonapartii*	−0.890	0.791	0.943	1.004
*A. devenanzii*	−0.002	1.140	−0.422	0.865
*A. balaenops*	−1.260	0.324	0.485	0.419

Mean and standard deviation (SD) for Dim1 and Dim2 for each species using all chirps (Fig. 4a) and with individual chirp averages within each species (Fig. 4b).

## Discussion

We found significant differences in chirp complexity and chirp structure variation across apteronotids. If the social complexity hypothesis applied to apteronotids, we expected that highly social species (*A. devenanzii* and *A. balaenops*) would have the most complex chirps and more variation in chirp structure, whereas highly territorial species (*P. hasemani*, *A. albifrons*, and *‘A.’ bonapartii*) would have simpler chirps and less variation in chirp structure. However, the variation in chirp complexity across the six species of apteronotids in this study could not be explained by group size. Territorial *P. hasemani* had the most complex chirps. While one social species, *A. devenanzii*, produced highly complex, multi-peaked chirps, the other highly gregarious species, *A. balaenops*, had very simple chirps. Territorial *A. albifrons* and *‘A.’ bonapartii* had relatively complex chirps, while semi-social *A. leptorhynchus* had simple chirps. These results are also inconsistent with a simple phylogenetic effect because chirp complexity varied substantially across species within the same genus. The genus *Adontosternarchus* contains both a species with highly complex chirps (*A. devenanzii*) and a species with very simple chirps (*A. balaenops*). Similarly, within *Apteronotus*, *A. albifrons* had more complex chirps than *A. leptorhynchus*. Variation in chirp structure across individual fish could not be explained by species group size either. The three species with the greatest variation in chirp structure included a highly territorial species (*P. hasemani*), a social species (*A. devenanzii*), and a semi-social species (*A. leptorhynchus*), while the species with the most stereotyped chirps (*A. balaenops*) is highly social. Two territorial species, *‘A.’ bonapartii* and *A. albifrons*, had intermediate variation in chirp structure. When controlling for variation within individuals, the structure of the average chirp produced by each individual still did not map onto social structure or genus. While gregarious *A. devenanzii* displayed substantial individual variation in chirp structure across fish, territorial *P. hasemani* and *‘A.’ bonapartii* also had substantial variation across individuals. Territorial *A. albifrons* and semi-social *A. leptorhynchus* had intermediate variation across individuals, and gregarious *A. balaenops* had very little individual variation in chirp structure. These data, therefore, do not support the social complexity hypothesis when sociality is measured as group size because neither chirp complexity nor variation in chirp structure was consistently associated with group size. These findings, however, do not rule out the possibility that social behavior plays a role in shaping signal structure in apteronotids.

The number of peaks and inflection points is partly confounded by duration. Longer chirps have more potential to contain multiple peaks than short chirps. When the number of peaks (and troughs) and inflections points was weighted by chirp duration, most species had similar complexity scores, except for *‘A.’ bonapartii*, which had short chirps with a relatively high number of peaks/troughs/IPs, and *P. hasemani*, which had simpler chirps relative to the other species when normalized to chirp duration. Frequency was often difficult to resolve in *P. hasemani* when EOD amplitude dropped below 15% of baseline (Turner et al. 2007) and peaks/troughs/IPs could not be measured during these periods. It is, however, unlikely that fish would be able to resolve variation in chirp complexity during periods of low amplitude anyways. The remarkably complex chirps of *P. hasemani* are, therefore, explained by their long durations, which were about eight times longer than those in the species with the next longest chirps, *A. devenanzii* and *A. albifrons*. We chose to present the results without normalizing for duration, however, given that chirp duration could be considered a component of complexity in and of itself. For example, if there was selection for more complex chirps, one way to achieve that might be by developing longer duration chirps that have more space for peaks, troughs, and inflections points. It is interesting that *‘A.’ bonapartii* produces both short and multi-peaked chirps, suggesting that duration alone cannot explain chirp complexity.

There was a male sampling bias in three of the six species (*A. leptorhynchus*, *A. devenanzii*, and *‘A.’ bonapartii*) included in this study, and sex was unknown in *A. balaenops*. Variation across species in sex ratio may represent a potential confound because chirp rate and structure are often sexually dimorphic in apteronotids. In *P. hasemani*, *A. leptorhynchus*,* A. devenanzii*, and *‘A.’ bonapartii*, males typically produce bigger and more multi-peaked chirps than females (Bastian et al. 2001; Zhou and Smith 2006; Ho et al. 2010; Petzold and Smith 2016). In two of these species, *P. hasemani* and *A. devenanzii*, we analyzed enough female chirps to confirm males had bigger and more complex chirps. In *‘A.’ bonapartii*, the one representative female also had less complex chirps, which is consistent with males having more multipeaked chirps than females (Ho et al. 2010). Because *A. leptorhynchus*,* A. devenanzii*, and *‘A.’ bonapartii* were mostly male, the complexity scores likely represent values close to the maximum for each species. *A. leptorhynchus* also produce two types of chirps. Small chirps are shorter in duration and lower in FM and are primarily produced to same-sex EODfs. Big chirps are longer with greater FM and are most often produced in response to opposite-sex EODfs (Kolodziejski et al. 2007). Male *A. leptorhynchus* chirp more than females and produce big chirps more often in response to large EODf differences (Bastian et al. 2001; Kolodziejski et al. 2007; Turner et al. 2007). The outliers in *A. leptorhynchus* in Fig. 4a were big chirps, which are more complex than small chirps. These relatively rare, big chirps may explain why chirp variation across chirps was inflated compared to Fig. 4b, where each fish’s chirps were averaged. Chirp structure, therefore, is still highly stereotyped across *A. leptorhynchus*. Females chirp much less than males in *A. leptorhynchus*, so it was more difficult to obtain recordings from females that had enough chirps to analyze. Even if more females were represented in the *A. leptorhynchus* sample, the average complexity score would likely be even lower, which would not affect interpretation of the results. In *A. albifrons* and *P. hasemani*, males typically produce longer chirps than females (Dunlap and Larkins-Ford 2003a, b; Kolodziejski et al. 2005; Petzold and Smith 2016). Our sample included roughly an equal number of male and female *A. albifrons* and *P. hasemani*. Chirp FM was similar across sexes in both species and chirp duration was comparable across sex in *A. albifrons*, but males had much longer chirps than females in *P. hasemani*. Accordingly, total complexity was higher in male *P. hasemani* but did not differ between the sexes in *A. albifrons*. If we only compared the males in these two species with the species where there was a male bias, our interpretations would be unaffected. *A. albifrons* chirp complexity is likely not sexually dimorphic. *P. hasemani* already had the most complex chirps, so chirp complexity would be even higher with only males included. Although sex was unknown in *A. balaenops*, there was very little individual variation in chirp structure across fish. It is, therefore, unlikely that there is significant sexual dimorphism in chirp structure in response to playback in this species. If the sample was heavily female biased or included reproductively immature fish, it is still possible the complexity scores calculated for *A. balaenops* here are lower than the natural species average.

This study only focused on chirp complexity and group size in six species from three genera, which does not provide sufficient sampling for a rigorous phylogenetic test of relationships between signaling and social structure. While chirp structure has been quantified in many other species not included here, their social behaviors are not well documented (Turner et al. 2007). We were, thus, limited to these six species where we have both chirp recordings and knowledge of their social structure. Despite our limited sample size, we still demonstrated extensive variation in chirp complexity within territorial and social species and within genera. *P. hasemani* had much more complex chirps than the other two territorial species, *A. albifrons* and *‘A’. bonapartii*. In addition, gregarious *A. devenanzii* had highly complex chirps, while the other social species, *A. balaenops*, had relatively simple, stereotyped chirps. The lack of a phylogenetic signal in chirp structure and/or complexity implies there is evolutionary lability in chirp signal features. Similarly, in *Liolaemus* lizards, phylogeny does not predict patterns of head bob display structure (Martins et al. 2004). In some closely related taxa with variation in complexity across functionally similar signals, however, the number of overlapping congeners predicts signal complexity or divergence (Seddon 2005; Nelson et al. 2022). Perhaps, the complex chirps of *A. devenanzii* evolved instead to aid in communicating species identity. Although more data on species distributions would be necessary to test this hypothesis, knifefish primarily rely on EOD frequency, and perhaps waveform, for species identification (Hopkins 1988; Kramer and Otto 1991; Dunlap and Larkins-Ford 2003a, b; Fugère and Krahe 2010). In addition, the fact that chirps are interactive signals that are typically only produced during social encounters suggests that they are primarily motivational signals, not signals of species identity.

Another important consideration is that the recordings in this study were collected in response to an artificial playback. Chirp rate is usually significantly higher during live behavioral interactions in the lab (Dunlap and Larkins-Ford 2003a, b; Freiler et al. 2022). Field studies have also revealed important, but overlooked, signaling interactions not produced in chirp chambers (Henninger et al. 2018). Signaling behavior would likely be more complex and representative of natural variation when captured in a dynamic social background. Indeed, for social species that aggregate in large groups, like *A. devenanzii* and *A. balaenops*, the chirp chamber paradigm may be a depauperate sensory environment. In Carolina chickadees, birds sing more complex songs in larger groups (Freeberg 2006). It is therefore possible that existing variation in chirp structure is not accurately captured with artificial playback experiments alone. One important advantage of using the chirp chamber paradigm, however, is the ability to accurately quantify chirp structure, which is challenging in recordings of live, interacting fish that are continuously moving relative to recording electrodes. The fine structure of chirps, which were analyzed in this study, would be exceptionally difficult to characterize during live interactions because of movement artifacts and social noise. We were, therefore, limited to using the highly controlled and uncontaminated recordings that can only be generated in response to artificial playback. Future studies, however, could examine how other aspects of chirping map onto social behavior using freely interacting fish. The temporal patterning of chirps can be complex and is often overlooked. In addition to chirp structure, chirp rate varies significantly across species (Turner et al. 2007). In species that have multiple chirp types, like *A. leptorhynchus*, the probability of chirp-type transitions can also vary across sex and social context (Oboti et al. 2025). Because most species do not have distinct chirp types, however, examining chirp-type transitions across a wide range species would not be possible. Chirps are also often produced in bouts and some species exhibit an ‘echo response’ whereby fish will chirp at a regular interval after perceiving a conspecific’s chirp (Zupanc et al. 2006; Henninger et al. 2018; Freiler et al. 2022). Interactive signal timing is an aspect of signal complexity that is often not explicitly compared across species with variation in social structure and is ripe for future investigation.

The relationship between signal complexity and sociality is not simple, and studies that have found a positive relationship between the two usually focus on a particular aspect of signal complexity, like repertoire size (Blumstein and Armitage 1997; McComb and Semple 2005; Freeberg 2006). Experimenters may also separate signal types that are not perceptually different to receivers or combine signal types that are functionally distinct to a receiver (Sturdy et al. 2000; Freeberg et al. 2012). Information theory metrics, such as Shannon’s entropy, can be used as a measure of signal complexity in species with distinct signal types (Da Silva et al. 2000; Kershenbaum et al. 2021; Nelson et al. 2022). Quantifying signal complexity, however, can be challenging in species with continuous graded variation in signal structure or in species where signal types overlap (Fischer et al. 2017; Kershenbaum et al. 2018; Keenan et al. 2020). While some species of electric fish have distinct chirp types, most species exhibit continuous variation in chirp FM and duration (Bastian et al. 2001; Kolodziejski et al. 2007; Turner et al. 2007). We chose to measure chirp complexity as the number of peaks/troughs and inflections points in this study, thereby allowing comparisons across all species. Complexity in electrocommunication, however, could also be manifest in other parameters. Many fish also produce more gradual EOD modulations with lower FM, including jamming avoidance responses, gradual frequency rises, rasps, warbles, and persistent EODf increases (Kramer 1987; Zakon et al. 2002; Petzold et al. 2018; Freiler et al. 2022). These modulations, however, are more infrequent and difficult to quantify, which is why we chose to focus primarily on chirp structure in this analysis.

Like our analysis, measures of social complexity have historically relied on simple metrics, like group size. Signal complexity, however, does not always cleanly map onto variation in group size, but may be related to a particular aspect of social behavior. Demographic roles and the frequency and nature of social interactions may play a bigger role than group size in driving variation in signal complexity (Pollard and Blumstein 2011; Sheehan and Tibbetts 2011; Aureli and Schino 2019). For example, songbird repertoires are larger in cooperatively breeding species (Leighton 2017). Signal complexity is also sometimes more tightly linked to aspects of mating system, which is not well understood in many electric fish species. In some species with larger sexual size dimorphism or stronger sexual selection, signal complexity is higher (Ord et al. 2001; Ord and Garcia-Porta 2012). In *Schizocosa stridulans* wolf spiders, females prefer males that produce more complex vibratory displays (Choi et al. 2022). In túngara frogs, males increase call complexity in response to competition, and females prefer complex calls (Rand and Ryan 1981). Sociality can be defined in terms of social networks, parental care, and mating systems, and newer approaches are integrating multiple measures of sociality or developing more quantitative ways to define social complexity (Avilés and Harwood 2012; Blumstein 2013; Hobson et al. 2019; Kappeler 2019; Rebout et al. 2021). Signal structure and complexity can also vary by type of social interaction. In eastern bluebirds and big brown bats, vocalization structure and syllable use varies with behavioral context and level of aggression (Gadziola et al. 2012; Rose et al. 2020). We relied primarily on group size and aggression to categorize the social behavior of the six species we used here. While we have personally worked with these species for many years and affiliation and agonism are critical aspects of a species’ social structure, we were limited to using the behaviors we can examine in a laboratory setting. More data on the social structures of electric fish species is needed to test the social complexity hypothesis across a broader phylogenetic scale. Future studies that examine more species in naturalistic settings will be important for determining how sociality and communication coevolve in apteronotids.

Species variation in signal function may also confound relationships between sociality and signal complexity. Chirps are used frequently in both same- and opposite-sex encounters, but their function can vary both within and across species. Chirps have been associated with both aggressive behaviors and submission (Hupé and Lewis 2008; Triefenbach and Zakon 2008; Bohorquez and Smith 2011; Silva et al. 2013; Freiler et al. 2022). While chirp structure has been well-characterized in response to playback in many species, chirp function is rarely studied outside of *A. leptorhynchus*. We are, therefore, limited in our ability to make predictions about why closely related species with similar social structures differ in chirp complexity. This confound is not unique to electric fishes, however. Many studies have not taken on the difficult task of identifying the specific social contexts in which all signals in a repertoire are produced (McComb and Semple 2005; May-Collado et al. 2007; Ord and Garcia-Porta 2012; Baeckens and Whiting 2021; Fichtel and Kappeler 2022). Still, divergence in species-specific signal functions could mask a positive relationship between signal complexity and sociality. In mormyrid electric fishes, it is the more solitary Clade A species that have greater variation in EOD waveform compared to shoaling Petrocephaline species (Hopkins 1980, 1981; Carlson et al. 2011; Baker et al. 2015; Carlson 2016). We might, however, expect differential selective pressures on signal elaboration in a courtship versus an aggressive social context. For example, greater waveform variation in Clade A species could suggest that sexual selection is stronger in more territorial species, which can drive the evolution of signal complexity in some cases (Rand and Ryan 1981; Ord et al. 2001; Ord and Garcia-Porta 2012). Alternatively, variation in signal structure may be more important for navigating frequent aggressive encounters in species with close or overlapping territories relative to a collective group with little agonism. The larger repertoire of cooperatively vs. noncooperatively breeding birds is driven primarily by a higher number of alarm and contact call types (Leighton 2017). More studies are beginning to focus on the functional variation in chirping across species (Batista et al. 2012; Zubizarreta et al. 2015; Freiler 2023; Perrone et al. 2024), which will provide the opportunity to ask whether chirp complexity varies across different social contexts.

One intriguing possibility is that chirps could be used as signals of individual identity in apteronotids. Individual recognition is linked to the evolution of complex signaling systems. In paper wasps and sciurid rodents, individual discrimination in visual and vocal signals is linked to greater sociality (Tibbetts 2002; Pollard and Blumstein 2011; Sheehan and Tibbetts 2011). Communicating individual identity can also be important during territorial contests (Bee et al. 2001). For example, territorial, pulse-type *Gymnotus* use individually distinctive EOD waveforms to discriminate between neighbors and other fish (McGregor and Westby 1992). It would be interesting to more thoroughly investigate natural social behavior of *A. devenanzii* and *‘A.’ bonapartii*, which have individually consistent and distinctive chirp structures that vary substantially across fish (Ho et al. 2010). There was more within-individual variation in chirp structure in *A. leptorhynchus*, as evidenced by the fact that when chirp parameters were averaged within individuals, the variance of chirp PCs decreased. Maybe in semi-social species with dominance hierarchies and more dynamic social relationships, it is more important to use intraindividual variation in signals to convey motivation. The need to signal individual identity versus other motivations may drive patterns of communicative complexity within and across species independently of group size or affiliation.

It is also possible that ecology impacts signal complexity and evolution, especially when signal transmission depends on abiotic features. Acoustic signal structure and frequency often vary with habitat complexity, ambient noise, and the risk of predation (Ryan 1985; Neuweiler 1989; Slabbekoorn and Smith 2002; Derryberry et al. 2020). Signal complexity is weakly linked to breeding range and migration in songbirds and to microhabitat in frogs (Ord and Garcia-Porta 2012). Specific foraging habitats can also explain some variation in repertoire size in songbirds (Leighton and Birmingham 2021). The distance over which a signal travels to reach a conspecific can influence display complexity in *Anolis* lizards (Nelson et al. 2022). The environment can shape EODs across gymnotiform fishes. Pulse-type gymnotiforms that inhabit low conductivity waters tend to have longer and thinner electric organs than species that live in high conductivity, to best match the resistance of the water and enhance the active space of EODs (Hopkins 1999). Predator abundance influences the evolution of EOD waveform by selecting for more symmetrical waveforms that are less detectable by electroreceptive predators (Stoddard 1999, 2002; Stoddard et al. 2019). While it is possible that environment influences chirp complexity in apteronotids, the very short distances over which electric signals travel may render natural selection on chirp structure weak relative to social or sexual selection. Social ecology could also influence signal design. In more species-dense areas, social noise from multiple species might create competition for signal space. African electric fishes have species-specific EODf ranges (Hopkins 1974). In the Amazon, however, EODf is not always a good indicator of species identity given the considerable overlap in EODf ranges between species (Kramer et al. 1981). Chirps are less likely to play a role in species identity relative to the static information contained in EODf and waveform (Hopkins 1988; Kramer and Otto 1991) and whether species density influences chirp design in apteronotids is unknown. Together, unknown features of mating system, dominance hierarchies, or behavioral ecology in apteronotids will more likely help account for the observed variation in chirp complexity across species.

Intuitively, social behavior and communication signals should be tightly linked during evolution because signals often function in mediating social interactions. The coevolutionary relationship between signal and social complexity, however, could later become obscured. Signal complexity can be lost due to stochasticity or high signaling costs even in the absence of relaxed selection (Ord et al. 2023). In toucans and barbets, behavioral and morphological traits related to visual displays coevolved tightly as display complexity increased, but individual signal components were secondarily lost independently (Miles and Fuxjager 2019). Logically, it is possible that social and signal complexity are codependent at early stages of their evolution but can later be separable. For example, social and signal complexity might have evolved together, with chirp complexity retained in *A. devenanzii*, but lost secondarily in *A. balaenops*. Perhaps losing signal complexity does not incur a large fitness cost in some social lineages. It is also possible some other aspect of signal complexity in *A. balaenops*, like rises or slower EODf modulations, map onto species group size instead of chirps. Testing these assumptions in knifefishes would require more behavioral data across Gymnotiformes to increase phylogenetic power. Moving forward, the social complexity hypothesis should be tested across multiple modalities and in different social and experimental contexts. Most likely, sociality is one of many driving forces of signal evolution and it may play a bigger role in explaining diversity in communication in some systems.

## Electronic supplementary material

Below is the link to the electronic supplementary material.

Supplementary Material 1

## Data Availability

Raw data on chirp parameters for each species are available on figshare (10.6084/m9.figshare.20493306).
