# Supplementary material for: Sociality does not predict signal complexity in response to playback in apteronotid weakly electric fishes
Source: Behav Ecol Sociobiol. 2025 Jul 10;79(7):78. doi: 10.1007/s00265-025-03619-y (PMC12246016; doi:10.1007/s00265-025-03619-y)
Supplement: Supplementary file 1 — Supplementary Material 1 [file 265_2025_3619_MOESM1_ESM.docx]

**Supplementary Material**

**Behavioral Ecology and Sociobiology**

**Sociality does not predict signal complexity in response to playback in apteronotid weakly electric fishes**

Megan K. Freiler^1,2^ and G. Troy Smith^1,2^

^1^Department of Biology, Indiana University, Bloomington, IN, USA

^2^Center for the Integrative Study of Animal Behavior, Indiana University, Bloomington, IN, USA

*Corresponding author information:*

Megan K. Freiler: e-mail: current - freil018@umn.edu, permanent - megan.k.freiler@gmail.com

Department of Entomology, University of Minnesota, St. Paul, MN

**Table S1** Chirp parameters by sex

|  | **PosFM**  **(Hz)** | **Duration**  **(s)** | **NegFM**  **(Hz)** | **Slopeup**  **(Hz ms^-1^)** | **Slopedown (Hz ms^-1^)** | **Peaks/**  **troughs** | **IPs** |
| --- | --- | --- | --- | --- | --- | --- | --- |
| **Male** |  |  |  |  |  |  |  |
| *P. hasemani* | 561.1±9.7 | 0.98±0.040 | N/A | 12.8±1.1 | 0.72±0.07 | 19.6±1.3 | 28.9±1.7 |
| *A. leptorhynchus* | 99.1±6.7 | 0.04±0.002 | 17.9±2.9 | 8.8±0.68 | 10.7±0.80 | 2.0±0.26 | 3.0±0.29 |
| *A. albifrons* | 242.4±12.7 | 0.14±0.007 | 3.1±0.17 | 21.3±1.4 | 2.5±0.20 | 4.8±0.48 | 10.6±0.82 |
| *‘A.’ bonapartii* | 252.3±14.6 | 0.06±0.003 | 4.6±0.30 | 21.1±1.1 | 13.9±1.3 | 5.3±0.35 | 8.5±0.44 |
| *A. devenanzii* | 173.1±5.7 | 0.17±0.011 | 3.1± 0.40 | 8.5±0.84 | 2.5±0.41 | 8.4±0.85 | 13.0±1.0 |
| *A. balaenops* | N/A | N/A | N/A | N/A | N/A | N/A | N/A |
| **Female** |  |  |  |  |  |  |  |
| *P. hasemani* | 562.3±17.1 | 0.61±0.062 | N/A | 21.8±1.7 | 1.5±0.22 | 12.8±0.90 | 20.4±1.4 |
| *A. leptorhynchus* | N/A | N/A | N/A | N/A | N/A | N/A | N/A |
| *A. albifrons* | 217.1±4.0 | 0.12±0.004 | 3.1±0.15 | 19.9±0.61 | 2.6±0.12 | 4.0±0.51 | 9.6±0.60 |
| *‘A.’ bonapartii* | 236.2±14.2 | 0.05±0.003 | 3.8±0.55 | 20.8±1.2 | 13.2±0.95 | 2.3±0.67 | 6.7±0.67 |
| *A. devenanzii* | 134.4±5.0 | 0.11±0.023 | 2.1±N/A | 7.8±0.86 | 5.0±0.87 | 4.0±1.4 | 6.2±1.6 |
| *A. balaenops* | N/A | N/A | N/A | N/A | N/A | N/A | N/A |

Mean±SEM for several chirp parameters across species separated by sex. N = total number of chirps. Males: *P. hasemani* (N=60), *A. leptorhynchus* (N=140), *A. albifrons* (N=53), *‘A.’ bonapartii* (N=50), and *A. devenanzii* (N=75). Females: *P. hasemani* (N=40), *A. albifrons* (N=60), *‘A.’ bonapartii* (N=3), and *A. devenanzii* (N=22). Only some chirps had a NegFM. Males: *P. hasemani* (N=0), *A. leptorhynchus* (N=80), *A. albifrons* (N=12), *‘A.’ bonapartii* (N=31), and *A. devenanzii* (N=4). Females: *P. hasemani* (N=0), *A. albifrons* (N=17), *‘A.’ bonapartii* (N=3), and *A. devenanzii* (N=1). IP = inflection point. All *A. leptorhynchus* were male. Sex was unknown in *A. balaenops*

**Fig. S1** Variation in chirp complexity across species when corrected for chirp duration. When divided by duration, the number of normalized **(a)** peaks (and troughs) (Kruskal-Wallis test: χ^2^=178.86, *P*<0.01) and **(b)** inflection points (Kruskal-Wallis test: χ^2^=246.69, *P*<0.01) in chirps varied across species but did not map onto sociality or genus. Territorial species are represented in green, semi-social in blue, and gregarious in purple. After normalizing for duration, *P. hasemani* (Pha, N=10) had less complex chirps than every other species while *‘A.’ bonapartii* (Abo, N=8) had relatively short but highly complex chirps. *A. albifrons* (Aal, N=13), *A. devenanzii* (Ade, N=15), *A. leptorhynchus* (Ale, N=14), and *A. balaenops* (Aba, N=14) had relatively similar chirp complexities when normalized to chirp duration. Letters denote significant pairwise differences from Wilcoxon rank-sum tests
